# Heterotopic Ossifications Following Intramedullary Stabilization of Femoral Fractures in Polytraumatized Patients

**DOI:** 10.3390/jcm13185557

**Published:** 2024-09-19

**Authors:** Gregor Wollner, Florian Hruska, Felix R. M. Koenig, Thomas Haider, Lukas L. Negrin

**Affiliations:** 1 Department of Orthopedics and Trauma-Surgery, Medical University of Vienna, 1090 Vienna, Austria; 2 Department of Biomedical Imaging and Image-Guided Therapy, Medical University of Vienna, 1090 Vienna, Austria

**Keywords:** polytrauma, heterotopic ossifications, femoral fracture, intramedullary stabilization, multiple traumas

## Abstract

Introduction: Heterotopic ossifications (HOs) are a well-known complication following total hip arthroplasty. Yet only little is known about the development of HOs following a femoral fracture and intramedullary stabilization in polytraumatized patients. Thus, the present study aimed to investigate whether the development of HOs is being observed more frequently in patients suffering polytrauma compared to those with single-extremity trauma. Materials and Methods: The retrospective outcome study was conducted at our level I trauma center. All patients admitted from 2010 to 2020 were included if they (1) presented with multiple injuries (≥2 body regions), (2) had an Injury Severity Score ≥16, (3) suffered a femoral fracture, and (4) were treated with intramedullary stabilization. Furthermore, a control group was established to match the polytrauma group (sex, age), who were suffering from single-extremity trauma (femoral fracture) which was treated with intramedullary stabilization. Subsequently, X-rays of the hip were performed and evaluated for up to one-year post-trauma. Results: Our study group consisted of 36 patients in total (91.7% male; mean age 39.4 ± 17.4 years, range: 18–82 years). The polytrauma (PT) group included 12 patients (mean age 39.5 years, median ISS 28), whereas the control group (single-extremity-trauma) included 24 patients (mean age 39.3 years). We documented HOs in nine (75%) patients in the PT group vs. five (20.8%) patients in the single-extremity group (*p* = 0.03). Conclusion: In this study, we were able to demonstrate that heterotopic ossifications are being observed significantly more frequently in patients suffering from polytrauma in comparison to patients with single-extremity trauma following intramedullary stabilization after a femoral fracture.

## 1. Introduction

Heterotopic ossifications (HOs) are defined by the presence of mature lamellar bone and bone marrow in soft tissues surrounding a major joint, mostly the hip joint [1,2]. HOs are known to be a common complication, especially following total hip arthroplasty, occurring in up to 40% [1,3,4]. However, HOs are not solely complications following total hip arthroplasty; they have been documented after pelvic and elbow surgery and in femoral fractures, thermal burns, and war-related explosive injuries [5,6]. Furthermore, there is evidence showing the development of HOs after a traumatic brain injury (TBI) or spinal cord injury (SCI), resulting in what we term neurogenic heterotopic ossifications (NHOs) [7]. It is reported that the prevalence of heterotopic ossifications after a central neurologic injury ranges from 10% to 53% [4]. There are multiple classifications that exist for the grading of heterotopic ossifications. However, the most common and established classification to determine the degree of ectopic bone formation in the hip was introduced by Andrew Brooker et al. in 1973 [8]. This classification divides the extent of HO formation into four classes ranging from islands of bone within the soft tissues about the hip to apparent bone ankylosis of the hip [8,9,10]. It is estimated that approximately 10% of all documented HO cases cause severe restrictions in joint motion or ankylosis [11].

To this day, the exact mechanisms behind the development of HOs remain unclear. However, certain risk factors have been identified, such as male gender, pre-existing ossifications, ankylosing spondylitis, age, and body mass index [3,12]. Recent studies have shown that even the selected surgical approach impacts the development of HO.

Even though extensive research has been conducted regarding the exact cellular mechanisms that promote HOs, the mechanisms are still poorly understood [5]. Inflammation induced by tissue injury is believed to be the major reason for the development of HOs [5]. It is believed that macrophages and mesenchymal stem cells play a crucial role at the cellular level [5].

The importance of clinical symptoms in the presence of HOs is typically low in most patients, especially in low-graded cases [1,10]. However, pain, limited range of motion, erythema, and swelling are observed more frequently in higher-graded HOs, which can take up to 6 months to present [4,10,13,14].

Treatment of HOs remains mostly prophylactic, with the administration of an NSAID to inhibit the osteogenic differentiation of progenitor cells [4]. Another well-described prophylactic approach to prevent HOs after a surgical procedure is radiation therapy within the first few days after surgery [2,12]. The prevalence of HOs after radiation therapy is reported to decrease by up to 50% [15]. However, surgical resection can be indicated in cases of advanced HOs, even though indication for surgery is usually reserved for patients with functional deficits as a result of the disorder (Brooker III-IV) [4,12,16].

Most publications have focused on the development of heterotopic ossifications after total hip arthroplasty, whereas only limited research has been carried out following multiple traumas, femoral fractures, and intramedullary stabilization. 

A study by Brumback et al. in the 1990s showed a prevalence of HOs after femoral fractures and consecutive intramedullary stabilization of 60% [17]. Other studies observed HOs in up to 54% of cases [18]. In addition, plate-osteosynthesis in polytrauma patients is associated with larger formations of symptomatic HO [19].

To our knowledge, no publication focuses on the development of HOs in polytraumatized patients following intramedullary stabilization of femoral fractures. Polytraumatized patients are an exceptional patient group because they suffer from multiple pathologies at the same time. Therefore, conclusions can be deceiving.

However, we observed several patients in our level I trauma center suffering from polytrauma and a femoral fracture and consecutively developing severe HOs after intramedullary stabilization in postoperative X-rays. The development of heterotopic ossifications in polytraumatized patients poses significant challenges to both diagnosis and management, often complicating their recovery and rehabilitation.

Thus, the present study aimed to investigate whether the development of HOs is being observed more frequently in patients suffering polytrauma in comparison to patients with single-extremity trauma.

## 2. Materials and Methods

### 2.1. Patients and Study Design

This retrospective outcome study received authorization from the Ethics Committee of the Medical University of Vienna (EK#1535/2024, approval date 4 June 2024) and adhered to the principles outlined in the Declaration of Helsinki. It is based on a data set routinely gathered at our level I trauma center from 2010 to 2020, including all admissions to the resuscitation room. All patients admitted from 2010 to 2020 were included if they (1) presented with multiple injuries (≥2 body regions), (2) had an Injury Severity Score (ISS) ≥16, (3) suffered a femoral fracture (intertrochanteric or shaft), and (4) were treated with intramedullary stabilization. In addition, a control group was established matching the polytrauma group based on the sex and age characteristics. Included in the control group were patients suffering single-extremity trauma (intertrochanteric- or femoral shaft fracture) who were treated with intramedullary stabilization. Subsequently, X-rays of the hip were evaluated up to one-year post-trauma in both groups, and HOs were classified by Brooker et al. [8].

### 2.2. Data Collection

The parameters for which data were collected included characteristics such as age, gender, ISS, Abbreviated Injury Scale (AIS) result for the different body regions, length of stay in the intensive care unit (ICU), and total length of hospital stay. The primary outcome measure was the development of HOs. All patients were followed up until one year following the trauma.

### 2.3. Evaluation of Heterotopic Ossifications

Standardized X-rays of the hip were performed at the date of admission and at 1,3, 6, and 12 months following trauma. All images were analyzed with PACS (Picture Archiving and Communication System), and one blinded investigator (F.H.) performed all measurements. Subsequently, the Brooker classification was applied.

### 2.4. Statistical Analysis

All statistical evaluations were performed utilizing the SPSS^®^ 26.0 software (SPSS Inc, Chicago, IL, USA). The Kolmogorov–Smirnow test was performed to evaluate the adherence of the parameters to a normal distribution. Parameters that conform to a normal distribution are expressed as mean ± standard deviation, whereas parameters that do not conform to a normal distribution are represented as median and interquartile range (IQR) in round brackets. Frequency counts and percentages characterize categorical data. They were analyzed using the χ^2^ test. Stacked bar charts were established to visualize results. In general, a *p*-value < 0.05 was considered significant. 

## 3. Results

### 3.1. Study Population

As shown in Table 1, our study group consisted of 36 patients (91.7% male; mean age 39.4 ± 17.4 years, range: 18–82 years). The polytrauma group (PT) included 12 patients (39.5 mean age, ISS 28), whereas the control group (single-extremity-trauma) included 24 patients (39.3 years of age). There was only one (8.3%) female patient in the PT group and consecutively two (8.3%) patients in the SE group. No gender-specific differences have been detected. 

### 3.2. Heterotopic Ossifications

**Figure 1:**
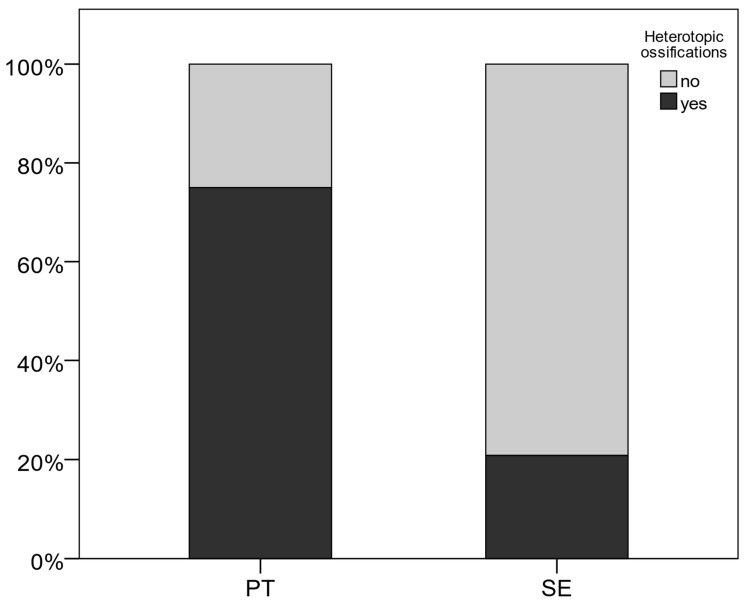
Occurrence of heterotopic ossifications.

We documented HO in nine (75%) patients in the PT group vs. five (20.8%) reported cases in the single-extremity group (*p =* 0.03). Figure 1 displays the occurrence of HO documented in the two cohorts. Furthermore, the odds ratio has been calculated to determine the association between group allocation and the occurrence of HO. It shows that the odds of developing HO in the polytrauma group are 11.4 times larger than in the single-extremity group.

**Figure 2:**
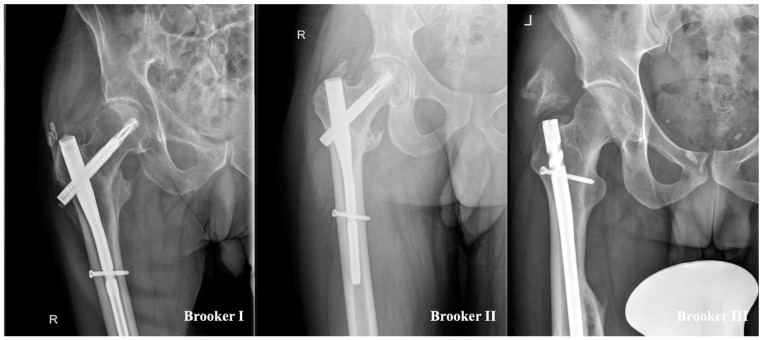
Display of different stages of heterotopic ossifications classified by Brooker et al. [8].

Figure 2 demonstrates the different stages of the Brooker classification in a standardized hip X-ray in the present study. In the polytrauma group, Brooker stage I and II were documented in four (33.3%) patients each, and stage III in only one (8.3%) patient. In the single-extremity cohort, Brooker stage I was documented three (12.5%) times and stage II two (8.3%) times. Brooker stage IV was not observed in the present study.

In the PT group, six (50%) intertrochanteric fractures and six (50%) shaft fractures were observed. The single-extremity group showed the following distribution: six (25%) intertrochanteric fractures vs. eighteen (75%) shaft fractures.

### 3.3. Polytrauma

Table 2 displays the PT cohort in detail. The mean stay at the ICU was documented with a median time of 11 days, whereas the median ISS was 28. No fatalities were observed. TBI was reported in three (25%) patients in the PT group, and all of them developed HO. SCI was observed in two (12.5%) patients, and both developed HO.

## 4. Discussion

The main finding of this study is that heterotopic ossifications are being observed more frequently in patients suffering from polytrauma in comparison to patients with single-extremity trauma, following intramedullary stabilization after a femoral fracture. The odds of polytraumatized patients developing HOs are more than eleven times larger than in the single-extremity group. To our knowledge, the present study is the first publication that has demonstrated this, thus, highlighting the phenomenon that patients suffering from multiple traumas are at particular risk for heterotopic ossifications. From our understanding, three main factors lead to extraordinarily high documentation of HO in polytraumatized patients, all in their own complex ways.

First, there is the local tissue trauma leading to the development of HO, which we believe is a similar pathophysiological mechanism compared to the single-extremity group. In the present study, all of the patients (PT and SE) suffered local tissue trauma resulting in a femoral fracture. HO development after trauma has been described by different authors and is believed to be induced by inflammation as a result of the pathologic recruitment of local and distant circulating cellular precursors [5,20,21]. Osteogenic precursors described in the development of heterotopic ossifications in recent years are Ctsk-Cre, Gli1-Cre, Wnt1-CreERT [20,22,23,24]. It has been shown that tissue that is prone to heterotopic ossification has an abnormally heightened or prolonged inflammatory response to injury [4]. Wong and Yu et al. hypothesize that trauma-induced heterotopic ossifications develop through endochondral osteogenesis, meaning cartilage formation occurs first, and then ossification is formed based on it [7,25]. As a matter of fact, patients suffering multiple traumas are in a hyperinflammatory state in the initial phases, which could stimulate the development of HO even more, eventually leading to severe joint restriction [26,27].

Secondly and even more importantly, a median length of stay at the Intensive Care Unit of 11 days, as well as a median hospital stay of 62.5 days, was documented in the PT group, meaning prolonged immobilization following surgical treatment. On the other hand, patients suffering singular femoral fractures following intramedullary stabilization are being mobilized in the first 24 h postoperatively in our level I trauma center. Immobilization is described as a positive predictor in the development of heterotopic ossifications [28,29,30]. Stoira et al. provided a detailed analysis of the occurrence of HO absent of trauma in a subset of 10 out of 52 patients (19%) who underwent extended mechanical ventilation as a result of a COVID-19 infection [31]. They hypothesized that prolonged immobilization resulting from longer sedation and neuromuscular blockade for severe ARDS has played a decisive role in heterotopic ossifications in their study [31]. Another possible pathophysiological mechanism for HOs in immobilized patients was described by Williams et al. in 2020 [32]. They observed immobilization-induced hypercalcemia leading to severe heterotopic ossifications and severe joint restrictions [32]. However, regardless of the exact pathomechanism, it is well known that immobilization is a risk factor for heterotopic ossifications.

Lastly, three (25%) patients suffered traumatic brain injury as well as spinal cord injury was documented in another two (16.7%) patients. Both factors are known to drive the development of heterotopic ossifications [7]. In the present study, all of the patients suffering from either TBI or SCI developed HOs. Garland et al. were the first authors to describe a correlation between head injuries and HOs in 1980 [33]. Later on, this phenomenon was described as neurogenic heterotopic ossifications (NHOs). It is believed that 20% of all patients suffering major central nervous system trauma develop NHOs [34]. It is known that patients with SCI and TBI have an altered serum that leads to the increased activity of osteoblasts, eventually leading to severe HOs on X-rays [34,35]. Even though an exact understanding of the cellular and molecular mechanisms of HOs’ formation, specifically in the context of neurotrauma, is lacking, Wong et al. provide a possible hypothesis [7]. They suggest that injury to the CNS triggers the release of osteogenic and inflammatory factors, such as SP, CGRP, OSM, IL-6, BMPs, and FGFs [7,34]. This cascade results in the proliferation of osteogenic and inflammatory mediators, thereby catalyzing the maturation of oligodendrocyte progenitor cells into fibroblasts and subsequently into chondrocytes, which experience hypertrophic changes and generate a cartilage matrix, eventually leading to the development of ectopic bone formation [7]. 

There are even hints of gender-specific differences in the expression of heterotopic ossifications after central nervous system trauma. Ranganathan et al. showed that male mice formed ~30% more ectopic bone when compared to female mice, possibly due to increased insulin-like growth factor-1 and bone morphogenetic protein (BMP) signaling in males [36]. Unfortunately, due to there being only one female patient in the PT group, gender-specific evaluations were not possible.

We think that all of the factors mentioned above contribute to the development of HOs in polytraumatized patients in their own way. In our opinion, there is a potential synergy of the mentioned pathophysiological mechanisms resulting in the impressive high number of documented heterotopic ossifications in polytraumatized patients following intramedullary stabilization and femoral fracture.

As previously discussed, heterotopic ossifications are not solely a radiological phenomenon. This abnormal development of bone tissue within soft tissue occurs due to diverse pathomechanisms and can cause severe symptoms, such as erythema, swelling, pain, and an increase and loss of the joint’s range of motion [14]. Even though we did not evaluate certain clinical parameters, such as pain or range of motion in the present study, numerous authors describe the occurrence of severe symptoms in Brooker III-IV cases, reserving indication for surgery for this particular group [3,12]. These advanced cases of heterotopic ossifications make recovery challenging for patients after total hip arthroplasty. However, we think that an already challenging recovery in polytraumatized patients suffering from advanced HOs, might prolongate or hinder their success. Therefore, we suggest follow-up X-rays of the hip joints regularly until 12 months postoperatively to detect patients at special risk, as 75% of all polytraumatized patients in the present study developed HOs. Even though patients at the ICU are being passively mobilized routinely, we agree with suggestions from Sun et al. that early treatment with a passive range of motion exercises should be implemented once the presence of HOs is confirmed to prevent possible ankylosing of joints [13]. Furthermore, if there are no contraindications, prophylaxis with NSAID should be considered for polytraumatized patients undergoing intramedullary stabilization after femoral fracture, as this has already been approved for standardized use in total hip arthroplasty. If there are known contraindications to pharmacological HO prophylaxis, external beam radiation should be considered as well. 

## 5. Limitations

The limitations of this study include the small sample size and the retrospective characteristic of the study over an extended period. Furthermore, the study population was limited to a single level I trauma center. Unfortunately, evaluating gender-specific differences was not possible in the present study due to the small cohort.

## 6. Conclusions

Heterotopic ossifications are being observed more frequently in patients suffering polytrauma in comparison to patients with single-extremity trauma, following intramedullary stabilization after femoral fracture. Clinicians working with this particular cohort, the polytraumatized patients, should be aware of the threat that their patients are possibly developing HOs, eventually even providing HO prophylaxis and conducting X-rays until 12 months postoperatively. We consider our paper a proof of concept that provides the basis for further research as part of prospective multicenter studies with a high number of patients. For further understanding, our aim will be to detect differences in expressed biomarkers between patients who developed HO versus patients who did not in the future.

## Figures and Tables

**Table 1:** Demographics and injury-related data.

	PT (*n* = 12)	Single Extremity (*n* = 24)	*p*-Value
Sex			
Male, *n* (%)	11 (91.7)	22 (91.7)	1.000
Female, *n* (%)	1 (8.3)	2 (8.3)	
Age (years)			
M ± SD	39.5 ± 17.6	39.3 ± 17.7	0.979
Occurrence of HO, *n* (%)	9 (75.0)	5 (20.8)	0.003
Brooker			
I, *n* (%)	4 (44.4)	3 (60.0)	
II, *n* (%)	4 (44.4)	2 (40.0)	
III, *n* (%)	1 (11.1)	0 (0.0)	
IV, *n* (%)	0 (0.0)	0 (0.0)	
Fracture type			
Femoral shaft, *n* (%)	6 (50.0)	6 (25.0)	0.157
Intertrochanteric, *n* (%)	6 (50.0)	18 (75.0)	

**Table 2:** Polytrauma-specific parameters.

Characteristics	PT (*n* = 12)
TBI, *n* (%)	3 (25.0)
Spinal cord injury, *n* (%)	2 (16.7)
Fatalities, *n* (%)	0 (0.0)
Stay at the ICU (days), median (IQR)	11.0 (14.8)
Length of hospital stay (days), median (IQR)	62.5 (57.3)
ISS, median (IQR)	28.0 (13.3)
AIS	
AIS head ≥3, *n* (%)	3 (25.0)
AIS abdomen ≥3, *n* (%)	5 (41.7)
AIS chest ≥3, *n* (%)	5 (41.7)
AIS face ≥3, *n* (%)	3 (25.0)
AIS extremities ≥3, *n* (%)	12 (100.0)
AIS external ≥3, *n* (%)	0 (0.0)

## Data Availability

The analyzed dataset in this study is available from the first author upon reasonable request.

## References

[1] Spinarelli A., Patella V., Petrera M., Abate A., Pesce V., Patella S. (2011). Heterotopic ossification after total hip arthroplasty: Our experience. Musculoskelet. Surg..

[2] Baird E.O., Kang Q.K. (2009). Prophylaxis of heterotopic ossification—An updated review. J. Orthop. Surg. Res..

[3] Willburger R.E., Brinkhoff F., Nottenkämper J., Krapp J., Oberberg S. (2022). Heterotopic ossification after total hip arthroplasty: When is development completed?. J. Orthop. Surg. Res..

[4] Ranganathan K., Loder S., Agarwal S., Wong V.W., Forsberg J., Davis T.A., Wang S., James A.W., Levi B. (2015). Heterotopic Ossification: Basic-Science Principles and Clinical Correlates. J. Bone Jt. Surg. Am..

[5] Cao G., Zhang S., Wang Y., Quan S., Yue C., Yao J., Alexander P.G., Tan H. (2023). Pathogenesis of acquired heterotopic ossification: Risk factors, cellular mechanisms, and therapeutic implications. Bone.

[6] Potter B.K., Forsberg J.A., Davis T.A., Evans K.N., Hawksworth J.S., Tadaki D., Brown T.S., Crane N.J., Burns T.C., O’Brien F.P. (2010). Heterotopic ossification following combat-related trauma. J. Bone Jt. Surg. Am..

[7] Wong K.R., Mychasiuk R., O’Brien T.J., Shultz S.R., McDonald S.J., Brady R.D. (2020). Neurological heterotopic ossification: Novel mechanisms, prognostic biomarkers and prophylactic therapies. Bone Res..

[8] Brooker A.F., Bowerman J.W., Robinson R.A., Riley L.H.J. (1973). Ectopic ossification following total hip replacement. Incidence and a method of classification. J. Bone Jt. Surg. Am..

[9] Hug K.T., Alton T.B., Gee A.O. (2015). Classifications in brief: Brooker classification of heterotopic ossification after total hip arthroplasty. Clin. Orthop. Relat. Res..

[10] Gautschi O.P., Cadosch D., Bauer S., Filgueira L., Zellweger R. (2008). Heterotopic ossification—From the aetiology to the current management. Unfallchirurg.

[11] Garland D.E. (1991). A clinical perspective on common forms of acquired heterotopic ossification. Clin. Orthop. Relat. Res..

[12] Łęgosz P., Otworowski M., Sibilska A., Starszak K., Kotrych D., Kwapisz A., Synder M. (2019). Heterotopic Ossification: A Challenging Complication of Total Hip Arthroplasty: Risk Factors, Diagnosis, Prophylaxis, and Treatment. BioMed. Res. Int..

[13] Sun E., Hanyu-Deutmeyer A.A. (2023). Heterotopic Ossification.

[14] Lawand J., Loeffelholz Z., Khurshid B., Barcak E. (2023). Heterotopic Ossification after Trauma. Orthop. Clin. N. Am..

[15] Bosse M.J., Poka A., Reinert C.M., Ellwanger F., Slawson R., McDevitt E.R. (1988). Heterotopic ossification as a complication of acetabular fracture. Prophylaxis with low-dose irradiation. J. Bone Jt. Surg. Am..

[16] Dey D., Wheatley B.M., Cholok D., Agarwal S., Yu P.B., Levi B., Davis T.A. (2017). The traumatic bone: Trauma-induced heterotopic ossification. Transl. Res..

[17] Brumback R.J., Wells J.D., Lakatos R., Poka A., Bathon G.H., Burgess A.R. (1990). Heterotopic ossification about the hip after intramedullary nailing for fractures of the femur. J. Bone Jt. Surg. Am..

[18] Juengteerapanich S., Udomkiat P., Mahaisavariya B. (2012). Heterotopic ossification after closed femoral nailing. J. Med. Assoc. Thai..

[19] Zeckey C., Hildebrand F., Mommsen P., Schumann J., Frink M., Pape H.-C., Krettek C., Probst C. (2009). Risk of symptomatic heterotopic ossification following plate osteosynthesis in multiple trauma patients: An analysis in a level-1 trauma centre. Scand. J. Trauma Resusc. Emerg. Med..

[20] Xu Y., Huang M., He W., He C., Chen K., Hou J., Huang M., Jiao Y., Liu R., Zou N. (2022). Heterotopic Ossification: Clinical Features, Basic Researches, and Mechanical Stimulations. Front. Cell Dev. Biol..

[21] Huang Y., Wang X., Zhou D., Zhou W., Dai F., Lin H. (2021). Macrophages in heterotopic ossification: From mechanisms to therapy. npj Regen. Med..

[22] Feng H., Xing W., Han Y., Sun J., Kong M., Gao B., Yang Y., Yin Z., Chen X., Zhao Y. (2020). Tendon-derived cathepsin K-expressing progenitor cells activate Hedgehog signaling to drive heterotopic ossification. J. Clin. Investig..

[23] Kan C., Chen L., Hu Y., Ding N., Li Y., McGuire T.L., Lu H., Kessler J.A., Kan L. (2018). Gli1-labeled adult mesenchymal stem/progenitor cells and hedgehog signaling contribute to endochondral heterotopic ossification. Bone.

[24] Olmsted-Davis E.A., Salisbury E.A., Hoang D., Davis E.L., Lazard Z., Sonnet C., Davis T.A., Forsberg J.A., Davis A.R. (2017). Progenitors in Peripheral Nerves Launch Heterotopic Ossification. Stem Cells Transl. Med..

[25] Yu T., Zhang J., Zhu W., Wang X., Bai Y., Feng B., Zhuang Q., Han C., Wang S., Hu Q. (2021). Chondrogenesis mediates progression of ankylosing spondylitis through heterotopic ossification. Bone Res..

[26] Guisasola M.C., Ortiz A., Chana F., Alonso B., Vaquero J. (2015). Early inflammatory response in polytraumatized patients: Cytokines and heat shock proteins. A pilot study. Orthop. Traumatol. Surg. Res..

[27] Mukhametov U., Lyulin S., Borzunov D., Ilyasova T., Gareev I., Sufianov A. (2023). Immunologic response in patients with polytrauma. Non-Coding RNA Res..

[28] Shehab D., Elgazzar A.H., Collier B.D. (2002). Heterotopic Ossification*. J. Nucl. Med..

[29] Meyers C., Lisiecki J., Miller S., Levin A., Fayad L., Ding C., Sono T., McCarthy E., Levi B., James A.W. (2019). Heterotopic Ossification: A Comprehensive Review. JBMR Plus.

[30] van de Langerijt O.N., Groot O.Q., Janssen M.M.A. (2024). Heterotopic Ossification of Bilateral Hips Post-COVID-19 and Prolonged Immobilization: A Case Report. JBJS Case Connect..

[31] Stoira E., Elzi L., Puligheddu C., Garibaldi R., Voinea C., Chiesa A.F. (2021). High prevalence of heterotopic ossification in critically ill patients with severe COVID-19. Clin. Microbiol. Infect. Off. Publ. Eur. Soc. Clin. Microbiol. Infect. Dis..

[32] Williams S.E., Forbush D. (2020). Immobilization Induced Hypercalcemia and Heterotopic Ossification in a Burn Patient [abstract]. PM&R.

[33] Garland D.E., Blum C.E., Waters R.L. (1980). Periarticular heterotopic ossification in head-injured adults. Incidence and location. J. Bone Jt. Surg. Am..

[34] Sullivan M.P., Torres S.J., Mehta S., Ahn J. (2013). Heterotopic ossification after central nervous system trauma: A current review. Bone Jt. Res..

[35] Cadosch D., Toffoli A.M., Gautschi O.P., Frey S.P., Zellweger R., Skirving A.P., Filgueira L. (2010). Serum after traumatic brain injury increases proliferation and supports expression of osteoblast markers in muscle cells. J. Bone Jt. Surg. Am..

[36] Ranganathan K., Peterson J., Agarwal S., Oluwatobi E., Loder S., Forsberg J.A., Davis T.A., Buchman S.R., Wang S.C., Levi B. (2015). Role of gender in burn-induced heterotopic ossification and mesenchymal cell osteogenic differentiation. Plast. Reconstr. Surg..

